# Pioglitazone Regulates Chondrocyte Metabolism and Attenuates Osteoarthritis by Activating Peroxisome Proliferator‐Activated Receptor Gamma

**DOI:** 10.1111/jcmm.70456

**Published:** 2025-02-26

**Authors:** Jiaqi Shi, Tianlun Gong, Yi Zhou

**Affiliations:** ^1^ Department of Orthopedics, Xiangyang Central Hospital Affiliated Hospital of Hubei University of Arts and Science Xiangyang Hubei People's Republic of China

**Keywords:** cartilage, chondrocytes, glycolysis, metabolic dysfunction, osteoarthritis, oxidative phosphorylation, pioglitazone, therapy

## Abstract

Osteoarthritis presents a significant clinical challenge due to its high prevalence and the resultant impairment of patients' motor function. Osteoarthritic chondrocytes are characterised by inflammation and metabolic disturbances. Pioglitazone, an agonist of peroxisome proliferator‐activated receptor γ (PPAR‐γ), has been demonstrated to exert anti‐inflammatory effects across various diseases. This study aims to investigate the potential protective effects of Pioglitazone on osteoarthritic chondrocytes. An in vitro chondrocyte inflammation model was established utilising IL‐1β. The impact of Pioglitazone on chondrocyte inflammation and extracellular matrix synthesis was evaluated through enzyme‐linked immunosorbent assay, immunofluorescence staining and Alcian blue staining. The affinity of Pioglitazone for PPAR‐γ was investigated using molecular docking techniques. Alterations in chondrocyte glycolysis and oxidative phosphorylation were examined using the Seahorse XF Analyser, and the influence of Pioglitazone on glucose uptake and the mitochondrial electron transport chain was further analysed. Pioglitazone was gavaged in a mouse OA model established by anterior cruciate ligament transection to evaluate the therapeutic efficacy of Pioglitazone. Our findings indicate that Pioglitazone mitigates chondrocyte inflammation and osteoarthritis in murine models by inhibiting the expression of inflammatory mediators such as TNF‐α, IL‐6 and PGE2, and by preventing the degradation of aggrecan and collagen II. Furthermore, Pioglitazone significantly upregulated the expression of glucose transporter 1 and stabilised the mitochondrial proton delivery chain in a PPAR‐γ‐dependent manner, thereby enhancing chondrocyte glucose uptake, glycolysis, and oxidative phosphorylation. These effects were partially reversed by the PPAR‐γ antagonist GW9662. Pioglitazone can confer chondroprotective benefits in osteoarthritis by activating PPAR‐γ.

## 1 Introduction

Osteoarthritis (OA) is a degenerative joint disease marked by chronic low‐grade inflammation of the joints, focal cartilage degradation, bone hypertrophy, synovial capsule thickening, and structural modifications of peri‐articular ligaments and peripheral muscles [1]. OA is the primary cause of disability among the elderly, impacting over 250 million individuals globally and imposing a substantial burden on healthcare systems [2]. Despite its prevalence, there remains a paucity of therapeutic agents capable of reversing the disease process in OA, necessitating joint replacement for patients with advanced stages of the condition to restore functionality [3, 4]. Therefore, there is a necessity for a comprehensive investigation into the disease mechanisms and potential intervention strategies for OA.

Presently, the risk factors for OA encompass aging, obesity, joint trauma, gender, and high‐intensity physical activity [5]. These mechanical, inflammatory, and metabolic factors contribute to the intricate pathogenesis of OA, resulting in chondrocyte dysfunction and joint destruction [6]. Epidemiological evidence further indicates that OA frequently coexists with metabolic disorders and comorbidities, such as diabetes and cardiovascular disease, which are often predictive of the rapid progression of OA [7]. The phenotype of OA associated with metabolic syndrome has been extensively characterised [8]. Emerging evidence indicates that common pathways implicated in both metabolic disease and OA, such as low‐grade inflammation and lipid metabolism, as well as metabolic factors exerting direct systemic effects on the joints, play a significant role [8]. Consequently, altered chondrocyte metabolism is linked to the OA phenotype associated with metabolic syndrome and may be a critical factor in the progression of other OA phenotypes.

Chondrocytes, the sole resident cells of articular cartilage, play a crucial role in maintaining cartilage function by facilitating the renewal of extracellular matrices, including aggrecan and collagen II [9]. Glucose serves as an essential energy substrate for chondrocytes and acts as a significant structural precursor for the synthesis of cartilage matrix components, such as aggrecan [10]. The glucose transporter protein Glucose transporter 1 (GLUT 1) is expressed in chondrocytes, and disruptions in glucose transport have been identified as a contributing factor to cartilage abnormalities in the development of OA [11]. Furthermore, there is increasing epidemiological evidence suggesting that diabetes mellitus constitutes an independent risk factor for OA. Numerous studies have demonstrated that diabetes exacerbates both the incidence and severity of OA [12, 13, 14]. Patients with OA who also have T2DM exhibit more severe clinical symptoms and structural changes in the joints compared to those without T2DM, indicating that diabetes is an independent predictor of severe OA [15]. Furthermore, the incidence of total joint arthroplasty is higher in the diabetic group than in the nondiabetic group, underscoring the undeniable strong correlation between diabetes mellitus and OA [16]. Animal studies have demonstrated that elevated glucose levels trigger inflammatory responses in mouse chondrocytes, induce apoptosis, suppress autophagy, and aggravate mitochondrial dysfunction in a dose‐dependent manner [17]. Furthermore, cartilage damage is more pronounced in T2DM mice following medial meniscus instability surgery [17]. However, the precise biomolecular mechanisms and pathogenesis by which diabetes mellitus compromises the integrity of osteoarthritic cartilage remain largely unexplored.

Pioglitazone, a thiazolidinedione antidiabetic medication, is commonly prescribed for patients with diabetes or metabolic syndrome [18]. Pioglitazone specifically activates peroxisome proliferator‐activated receptor‐γ (PPAR‐γ), thereby enhancing cellular sensitivity to insulin [19]. This activation leads to increased glucose metabolism and mitochondrial activity in the context of neuroinflammatory and neurodegenerative diseases [20]. Additionally, Pioglitazone has been shown to promote glycolysis and oxidative phosphorylation in mice subjected to a high‐fructose diet [21]. However, its effects on OA and chondrocytes remain unexplored.

In the present study, we investigated the impact of Pioglitazone on chondrocyte metabolism and examined the underlying mechanisms using PPAR‐γ inhibitors. Additionally, the therapeutic effects of Pioglitazone were investigated through intra‐articular injection of either Pioglitazone or an equivalent volume of saline in a mouse model of OA.

## 2 Methods

### 2.1 Primary Chondrocyte Extraction

Seven‐week‐old C57BL/6J mice were procured from Shulaibao (Wuhan) Biotechnology Co. All experimental procedures were conducted in strict accordance with the guidelines for animal experimentation as approved by our Institutional Animal Care and Use Committee. The mice were euthanised under isoflurane anaesthesia, and the knee joints were exposed in a sterile environment. The articular cartilage was subsequently dissected and isolated, followed by an eight‐hour digestion at 37°C on a constant temperature shaker in DMEM/F12 medium containing 0.2% type II collagenase. The digested cartilage tissue was filtered through a sterile filter and centrifuged at 1200 rpm for 8 min to extract primary chondrocytes. The chondrocytes were cultured in a constant temperature incubator set at 37°C with 5% carbon dioxide, using a DMEM/F12 medium supplemented with 10% fetal bovine serum and 1% penicillin–streptomycin. The culture medium was replenished every 3 days. Upon reaching 90% confluency, the chondrocytes were digested with trypsin and passaged. To ensure phenotypic stability, only second to third passage chondrocytes were utilised for subsequent experiments.

### 2.2 Enzyme‐Linked Immunosorbent Assay (ELISA)

Interleukin‐1β (IL‐1β), a pivotal pro‐inflammatory cytokine, enhances the expression of inflammatory mediators, including tumour necrosis factor‐alpha (TNF‐α), interleukin‐6 (IL‐6), and prostaglandin E2 (PGE2), within chondrocytes, thereby exacerbating local inflammatory responses [22]. Concurrently, PGE2 further stimulates the expression of matrix metalloproteinases, leading to the degradation of the extracellular matrix [23]. Consequently, to evaluate the inflammatory status of chondrocytes, concentrations of IL‐6, TNF‐α and PGE2 in mouse chondrocyte supernatants were quantified using specific ELISA kits.

### 2.3 Molecular Docking

To evaluate the binding affinities and interaction modes between the drug candidate and its targets, the in silico protein–ligand docking software Autodock Vina 1.2.2 was utilised [22]. The molecular structure of Pioglitazone was obtained from the PubChem Compound database (https://pubchem.ncbi.nlm.nih.gov/) [24]. The 3D coordinates of PPAR‐γ were downloaded from the PDB (http://www.rcsb.org/pdb/home/home.do). For the docking analysis, all protein and molecular files were converted into PDBQT format, excluding all water molecules and incorporating polar hydrogen atoms. The grid box was centered to encompass the domain of each protein, allowing for unrestricted molecular movement. The dimensions of the grid box were set to 30 Å × 30 Å × 30 Å, with a grid point spacing of 0.05 nm. Molecular docking studies were conducted using Autodock Vina version 1.2.2 (http://autodock.scripps.edu/).

### 2.4 Detection of Mitochondrial Respiration Rate by XF Analyser

The XF24 extracellular flux analyser was utilised to evaluate mitochondrial oxygen consumption. Chondrocytes were first adhered to the bottom of each well, after which Pioglitazone was administered to the designated wells in accordance with the experimental protocol and incubated for 24 h. Subsequently, inhibitors of mitochondrial respiratory complexes III (antimycin A; 1 μM) and V (oligomycin; 1 μM), along with the mitochondrial oxidative phosphorylation uncoupler carbonylcyanide‐4‐(trifluoromethoxy) phenylhydrazone (FCCP; 2 μM), were sequentially introduced to the wells following the programmed protocols. FCCP, a mitochondrial protonophoric uncoupler, disrupts the proton gradient across the inner mitochondrial membrane, thereby rapidly increasing oxygen consumption when applied to cells [25]. Subsequently, the oxygen consumption rate (OCR) and extracellular acidification rate (ECAR) were measured.

Glycolysis was evaluated using the Glycolytic Stress Test Kit (Agilent, Santa Clara, CA). Specifically, the area under the curve (AUC) of the extracellular acidification rate (ECAR) was calculated between the applications of glucose and oligomycin. The competitive glucose inhibitor 2‐Deoxy‐D‐glucose (2‐DG) was employed to inhibit glycolysis. Basal respiration, which provides ATP synthesis, was assessed, and proton leak was calculated as the AUC of the oxygen consumption rate (OCR) prior to oligomycin treatment. An increase in basal respiration corresponds to an increase in the ATP turnover rate, and vice versa. Oligomycin functions as an inhibitor of ATP synthase. Therefore, the AUC of the OCR and the interval between oligomycin and FCCP treatments were utilised to define the ATP production capacity of the cells. Respiratory capacity, indicative of the maximal activity of electron transport and oxidative phosphorylation, was determined by calculating the AUC of the OCR prior to antimycin A treatment and subsequent to FCCP treatment.

### 2.5 Measurement of Intracellular ATP Concentration

Intracellular ATP concentration was quantified using an ATP colorimetric assay kit (K354; Biovision). Upon reaching a cell density of 5 × 10^5^, chondrocytes were lysed with 120 μL of lysis buffer (C3228; Merck). Twenty microliters of cell lysate were incubated with the ATP reaction mixture for 30 min, and ATP levels were subsequently measured at an absorbance of 570 nm. Protein concentrations were quantified using the Bradford assay, and the resulting values were normalised to the protein concentration of the sample.

### 2.6 Glucose Uptake Assay

Fresh medium was introduced to chondrocytes following 24 h of treatment with either the carrier or Pioglitazone. To evaluate glucose uptake, the fluorescent glucose analog 2‐[N‐(7‐nitrobenzo‐2‐oxa‐1,3‐diazol‐4‐yl)amino]‐2‐deoxyglucose (2‐NBDG; 2760,1 mmol/L; Biovision, Milpitas, CA) was incubated with either the carrier or insulin (1 μM) for 30 min after an overnight starvation period. Green fluorescence signals were visualised using an FV10i confocal laser scanning microscope (Olympus, Tokyo, Japan).

### 2.7 Mitochondrial Respiratory Enzyme Activity Assay

Enzyme activities associated with the mitochondrial respiratory chain were quantified in chondrocyte‐rich cultures employing a thermostatically regulated ThermoSpectronic spectrophotometer (Thermo Fisher Scientific). For all enzyme activity assays, a minimum of one replicate was conducted for each sample, and the activity was quantified as nanomoles per minute per microgram of protein. All reagents utilised in the enzyme assays were procured from Sigma‐Aldrich.

To assess the activity of nicotinamide adenine dinucleotide (NADH) cytochrome c reductase (NCCR; complexes I and III), chondrocyte homogenates (20 μg of protein) were incubated in a phosphate buffer (pH 7.4) containing 1.5 mM KCN and 1 mM β‐NADH, within a mixture comprising 50 mM K for 2 min at 37°C. Following this incubation, the reduction of oxidised cytochrome c was initiated by the addition of 0.1 mM cytochrome c and monitored by measuring absorbance at 550 nm for 3 min at 37°C. The molar extinction coefficient of cytochrome c at 550 nm was determined to be 18,500 M/cm.

For the assessment of succinate cytochrome c reductase (complex II + III) activity, chondrocyte homogenates (30 μg) were incubated in a 40 mM K_2_HPO_4_ buffer (pH 7.4) containing 1.5 mM KCN and supplemented with 20 mM succinate. Following a 5‐min equilibration period at 37°C, cytochrome c was added to a final concentration of 50 μM, and the reaction was monitored at 550 nm for 3 min at 37°C.

The activity of cytochrome c oxidase (CCO; complex IV) was determined by measuring the oxidation of reduced cytochrome c, with the primary rate constant used as the measure of CCO activity. The activity was quantified as a first‐order rate constant, derived from the known concentration of iron cytochrome c and the enzyme quantity in the assay mixture. Chondrocyte homogenates (30 μg) and 500 mM K2HPO4 (pH 7.4) were preincubated for 5 min at 30°C. The reaction was initiated by the addition of ferric cytochrome c (final concentration 45 μM) and monitored at 550 nm for 3 min at 30°C. The background rate was determined following the addition of 1.0 μM K_3_Fe(CN)_6_.

### 2.8 Measurement of Lactate Concentration

Prior to the assay, each group of cultured chondrocytes was washed three times with PBS and incubated with fresh medium for 30 min. Subsequently, the culture medium was collected for extracellular lactate analysis using the Lactate Colorimetric Assay Kit (K627; Biovision). Primary chondrocytes (5 × 10^5^) were harvested and incubated in 120 μL of lysis buffer (C3228; Merck). A 1 μL aliquot of the culture medium was then incubated with the lactate reaction mixture for 30 min. Lactate concentrations were measured by absorbance at 450 nm. The obtained values were normalised to the reaction volume and the protein concentration of the sample, with the protein concentration determined via the Bradford assay.

### 2.9 Measurement of Intracellular Glycogen Concentration

The glycogen concentration in chondrocytes (5 × 10^5^) was quantified using the Glycogen Colorimetric Assay Kit II (K648; Biovision). Twenty microliters of cell lysate were incubated with the glycogen reaction mixture for 30 min. Glycogen levels were subsequently measured at an absorbance of 450 nm. To account for variability in cell counts across samples, protein concentration was determined to estimate cell numbers, assuming a relatively constant protein content per cell. The obtained values were normalised to the reaction volume and protein concentration of the sample, with protein concentration determined via Bradford analysis.

### 2.10 Quantitative Real‐Time Fluorescence PCR (RT‐qPCR)

RT‐qPCR was conducted using a Roche LightCycler 480 instrument (Roche Applied Science, Mannheim, Germany). Ten nanograms of DNA were combined with 10 μL of LightCycler 480 SYBR Green I premix (Roche Applied Science), which included forward and reverse primers at a final concentration of 0.4 μM (5 μmol). The total reaction volume was adjusted to 20 μL. The quantitative PCR protocol was executed as follows: (1) Denaturation: an initial incubation at 50°C for 2 min, followed by denaturation at 95°C for 1 min; (2) Amplifications: 15 s at 95°C, annealing at 60°C for 20 s, extension at 72°C for 15 s, repeat for 40 cycles; (3) Dissolution curve: the dissolution curve was generated by the built‐in software of the PCR Amplifier. The cDNA and PCR products were stored at 4°C. Each quantitative PCR was conducted independently in triplicate, with relative abundance calculated using the formula Δ*Ct* = [*Ct* (target gene) − *Ct* (β‐actin)]; ΔΔ*Ct* = [Δ*Ct* (sample) − Δ*Ct* (control)]. The quantitative gene expression is expressed as 2^−ΔΔ*Ct*^, representing the copy number of mitochondrial DNA in each sample. Values were determined for each quantitative PCR run, with β‐actin serving as the control for normalisation.

### 2.11 Immunofluorescence

Subsequently, methanol‐fixed chondrocytes were washed with PBS buffer and permeabilised using 0.1% Triton X‐100 in 0.1% sodium citrate. The cells were then incubated with anti‐GLUT1 (1:250; ab115730, abcam), anti‐Aggrecan (1:2000; ab315486, abcam), anti‐Collagen II (1:200; ab34712, abcam), or anti‐PPAR‐γ (1:100; ab272718, abcam) in 10% goat serum PBS buffer. Chondrocytes were incubated with the appropriate fluorescence‐conjugated secondary antibody (Thermo Fisher Scientific) for 1 h. Fluorescence signals were captured using an FV10i confocal laser scanning microscope (Olympus, Tokyo, Japan). Quantitative analysis of immunofluorescence results was performed using ImageJ software. The image was converted to grayscale, a threshold was set to identify the stained area, and the staining intensity value was calculated.

### 2.12 Alcian Blue Staining

Alcian blue staining was employed to assess the glycosaminoglycans synthesised by chondrocytes, revealing a positive correlation between staining intensity and aggrecan secretion [26]. Alcian Blue staining was conducted utilising the Alcian Blue Staining Kit (Vector, H‐3501). In brief, sections were incubated in Alcian Blue solution (pH 2.5) for 30 min and subsequently restained with Nuclear Solid Red, following the manufacturer's instructions. Imaging of the sections was performed using a Zeiss Axioplan2 brightfield microscope equipped with a Zeiss Axiocam HRc colour camera. For quantitative analysis using ImageJ software, the procedure involved selecting the region of interest, converting the image to grayscale, setting a threshold to identify the stained areas, and calculating the staining intensity value.

### 2.13 Establishment of OA Mouse Model

Twelve‐week‐old male C57BL/6J mice, with an average weight of 25 ± 2 g, were procured from Shulaibao (Wuhan) Biotechnology Co. The mice were maintained under specific pathogen‐free conditions, adhering to a 12‐h light/dark cycle, and were provided with ad libitum access to food and water. All animal experiments were performed in compliance with the Guide for the Care and Use of Laboratory Animals and received approval from the Ethics Committee of Renmin hospital of Wuhan University. The OA model was established via anterior cruciate ligament transection (ACLT) according to previous research [27, 28], followed by daily intra‐articular injections of Pioglitazone or an equivalent volume of saline at 12:00 AM. The mice were divided into four groups: Sham, ACLT, ACLT + 10 mg/kg Pioglitazone, ACLT + 20 mg/kg Pioglitazone. The sham mice underwent a similar incision on the right joint without compromising the ligaments. At 8 weeks postoperatively, all mice were anaesthetised with isopentane to minimise pain, and subsequently euthanised via cervical dislocation. The knee joints of mice were harvested for subsequent analyses.

### 2.14 Haematoxylin and Eosin (H&E) and Safranin O‐Fast Green Staining

The knee joints were fixed in 4% paraformaldehyde, decalcified using ethylenediaminetetraacetic acid (EDTA), dehydrated through a graded series of ethanol solutions, and embedded in paraffin. Following deparaffinisation and rehydration, 5 μm sections were stained with haematoxylin (H8070; Solarbio) for 5 min and eosin (A600190; Sangon) for 3 min. For Safranin O‐Fast Green staining, tissue sections were incubated in Safranin O for 5 min, followed by staining with a fast green solution for 1 min. Images were captured using an Olympus BX53 microscope (Olympus, Japan). A semi‐quantitative histologic scoring system (0–6 scale) established by the OA Research Society International (OARSI) was employed to evaluate the images and estimate cartilage damage.

### 2.15 Immunohistochemical (IHC) Staining

Following deparaffinisation and rehydration, tissue sections were immersed in a citrate‐buffered antigen retrieval solution and subjected to heat‐induced antigen retrieval, followed by incubation to block endogenous peroxidase activity. After blocking with 1% BSA, the sections were incubated overnight at 4°C with a primary antibody anti‐Aggrecan (1:2000; ab315486, abcam), anti‐Collagen I (1:100; ab270993, abcam) anti‐Collagen II (1:200; ab34712, abcam), anti‐PPAR‐γ (1:100; ab272718, abcam), or anti‐GLUT1 (1:250; ab115730, abcam). This was followed by a 1‐h incubation at 37°C with an HRP‐labelled goat anti‐rabbit IgG (#31460; 1:500, ThermoFisher) secondary antibody for 1 h at 37°C. The sections were then stained with 3,3′‐diaminobenzidine (DAB; DAB‐1031; Maxim) counterstained with haematoxylin before being mounted and imaged using a BX53 microscope (Olympus). For Aggrecan, Collagen I and Collagen II, the integrated optical density and area of interest of all positive staining were quantified [29]. The sectional areas of articular cartilage were determined by calculating the integrated optical density per unit area of interest to analyse the extracellular matrix. For PPAR‐γ and GLUT1, positive cells were identified using ImageJ software to compute the ratio of positive cells to total cells.

### 2.16 Data Analysis

All values are expressed as mean ± standard deviation (SD). For biochemical experiments involving multiple groups, the group means were assessed using one‐way ANOVA, followed by the Scheffé multiple range test for post hoc analysis of individual means. Statistical analyses were conducted using GraphPad Prism version 8.0. A *p* < 0.05 was considered statistically significant for both the control and IL‐1β groups.

## 3 Results

### 3.1 Pioglitazone Reverses IL‐1β‐Induced Chondrocyte Phenotypic Loss

**FIGURE 1:**
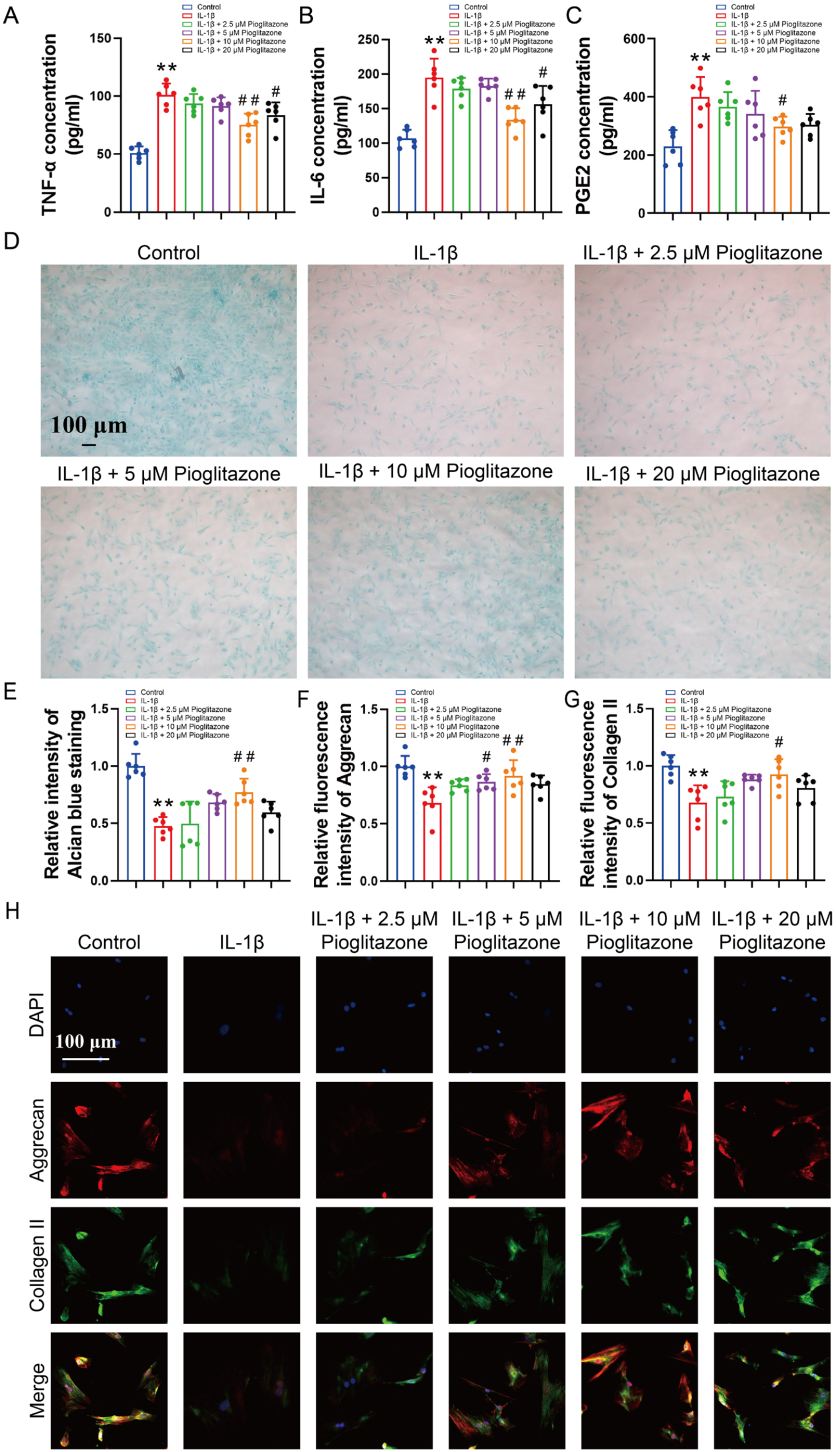
Pioglitazone reverses IL‐1β‐induced chondrocyte phenotypic loss. (A–C) Concentrations of TNF‐α, IL‐6 and PGE2 in chondrocyte supernatants were detected by ELISA. Pioglitazone was observed to reduce the levels of TNF‐α, IL‐6 and PGE2 in the chondrocyte culture medium. (D, E) Intensity of staining of chondrocytes was detected by Alcian blue staining to assess aggrecan expression and quantified through the ImageJ software. Pioglitazone partially restored the intensity of Alcian staining in IL‐1β‐induced chondrocyte. (F–H) Expression of aggrecan and collagen II in chondrocytes was assessed and quantified by immunofluorescence staining. A concentration of 10 μM Pioglitazone restored the fluorescence intensity of aggrecan and collagen II in IL‐1β‐induced chondrocytes. Each data point represents a measurement. Values are means ± SD of analyses through the one‐way ANOVA test (*n* = 6). *p < 0.05, **p < 0.01, versus control group; ^#^ *p* < 0.05, ^##^ *p* < 0.01 versus IL‐1β group.

Based on the intervention concentrations commonly used in previous studies, chondrocyte inflammation was induced using 10 ng/mL of interleukin‐1 beta (IL‐1β) [30]. ELISA results demonstrated that IL‐1β significantly elevated the levels of TNF‐α, IL‐6, and PGE2 in the chondrocyte culture medium. This increase was partially mitigated by the administration of Pioglitazone (Figure 1A–C). Furthermore, Pioglitazone was found to counteract the IL‐1β‐induced phenotypic loss in chondrocytes (Figure 1A–C). The secretion of aggrecan and collagen II to maintain extracellular matrix homeostasis in cartilage tissues is a primary function of chondrocytes. Alcian staining is indicative of the chondrocytes' capacity to secrete aggrecan. Results from Alcian staining demonstrated that IL‐1β reduced the staining intensity in chondrocytes compared to control cells, whereas Pioglitazone partially restored the staining intensity, with the most pronounced effect observed at a concentration of 10 μM (Figure 1D,E). Immunofluorescence staining revealed that IL‐1β reduced the fluorescence intensity of Aggrecan and collagen II in chondrocytes, whereas treatment with 10 μM Pioglitazone restored the fluorescence intensity (Figure 1F–H). These findings suggest that Pioglitazone mitigates IL‐1β‐induced inflammation and preserves the cartilage phenotype in chondrocytes. Consequently, a concentration of 10 μM Pioglitazone was employed in subsequent experiments.

### 3.2 Pioglitazone Exerts Chondrocytoprotective Effects Through PPAR‐γ

**FIGURE 2:**
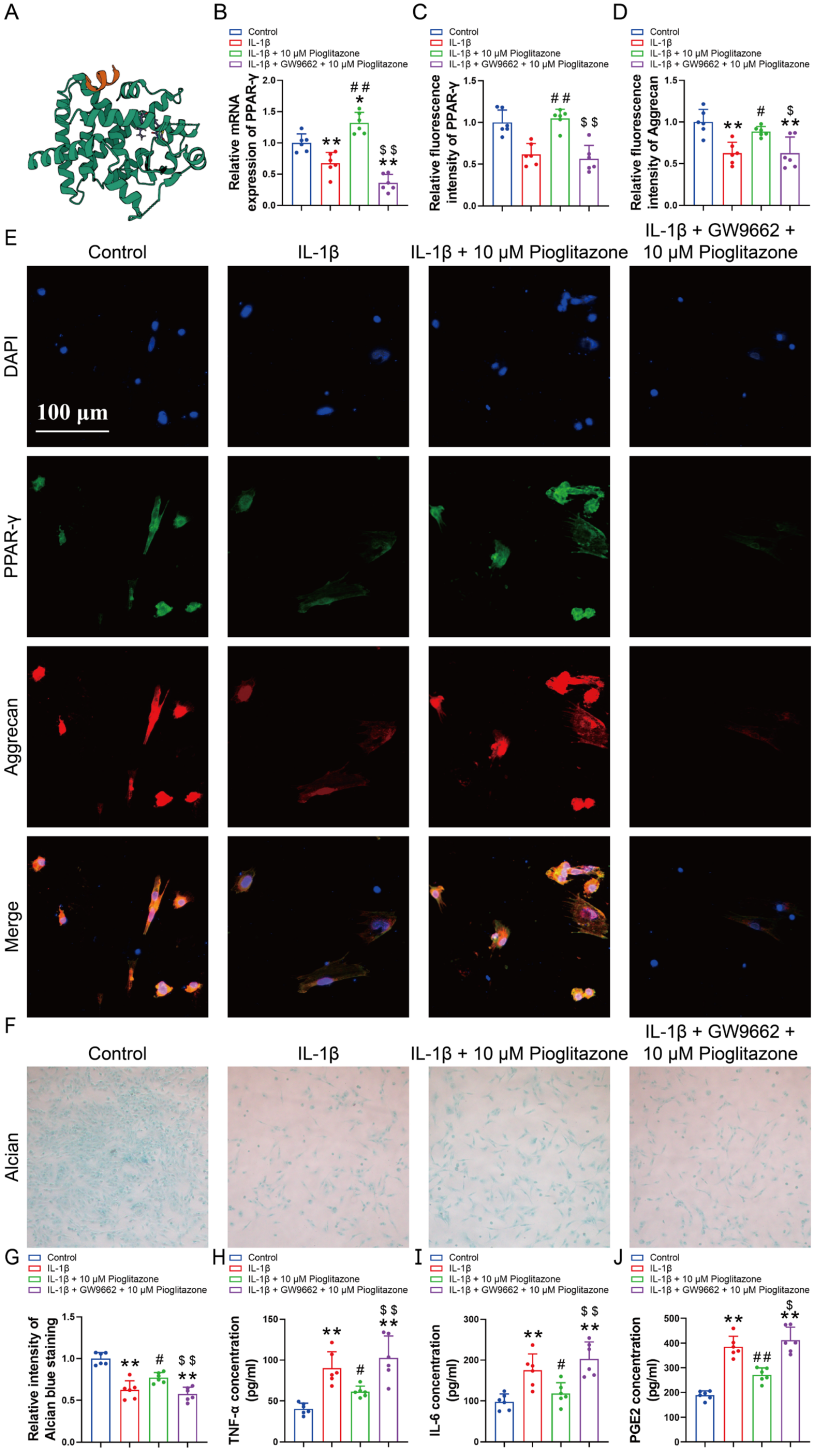
Pioglitazone exerts chondrocytoprotective effects through PPAR‐γ. (A) Molecular docking studies demonstrated that Pioglitazone exhibits a low binding energy of −9.25 kcal/mol with PPAR‐γ. (B) The RT‐qPCR analysis demonstrated that Pioglitazone failed to enhance the mRNA expression of PPAR‐γ in chondrocytes subjected to GW9662 treatment. (C–E) Expression of PPAR‐γ and Aggrecan in chondrocytes was assessed and quantified by immunofluorescence staining. The fluorescence intensity of PPAR‐γ and Aggrecan was diminished following GW9662 intervention. (F, G) Intensity of staining of chondrocytes was detected by Alcian blue staining to assess aggrecan expression and quantified through the ImageJ software. Pioglitazone augmented the intensity of Alcian blue staining, the effects of Pioglitazone were nullified by GW9662 intervention. (H–J) Concentrations of TNF‐α, IL‐6 and PGE2 in chondrocyte supernatants were detected by ELISA. GW9662 was observed to elevate the concentrations of TNF‐α, IL‐6, and PGE2 in the chondrocyte culture medium. Each data point represents a measurement. Values are means ± SD of analyses through the one‐way ANOVA test (*n* = 6). *p < 0.05, **p < 0.01, versus control group; ^#^ *p* < 0.05, ^##^ *p* < 0.01 versus IL‐1β group; ^$^ *p* < 0.05, ^$$^ *p* < 0.01 versus IL‐1β + 10 μM Pioglitazone group.

Given that Pioglitazone is a well‐established PPAR‐γ agonist, we utilised molecular docking to assess its binding affinity to PPAR‐γ. The molecular docking analysis indicated that Pioglitazone exhibits a low binding energy of −9.25 kcal/mol with PPAR‐γ, suggesting a highly stable interaction (Figure 2A). Subsequently, we conducted an in‐depth investigation into the mechanism of action of Pioglitazone utilising the PPAR‐γ antagonist GW9662. RT‐qPCR analysis revealed that Pioglitazone upregulated the expression of PPAR‐γ in chondrocytes, whereas GW9662 intervention significantly reduced PPAR‐γ mRNA expression (Figure 2B). Additionally, Pioglitazone augmented the fluorescence intensity of PPAR‐γ and Aggrecan in IL‐1β‐treated chondrocytes and increased the intensity of Alcian blue staining (Figure 2C–G). Notably, these effects induced by Pioglitazone were abrogated following GW9662 intervention (Figure 2C–G). In addition, Pioglitazone was unable to mitigate the up‐regulation of TNF‐α, IL‐6, and PGE2 concentrations induced by IL‐1β in chondrocytes treated with GW9662 (Figure 2H–J). These findings suggest that Pioglitazone may exert chondrocytoprotective effects in OA through the activation of PPAR‐γ.

### 3.3 Pioglitazone Reversed the Suppression of Chondrocyte Glycolysis Induced by IL‐1β

**FIGURE 3:**
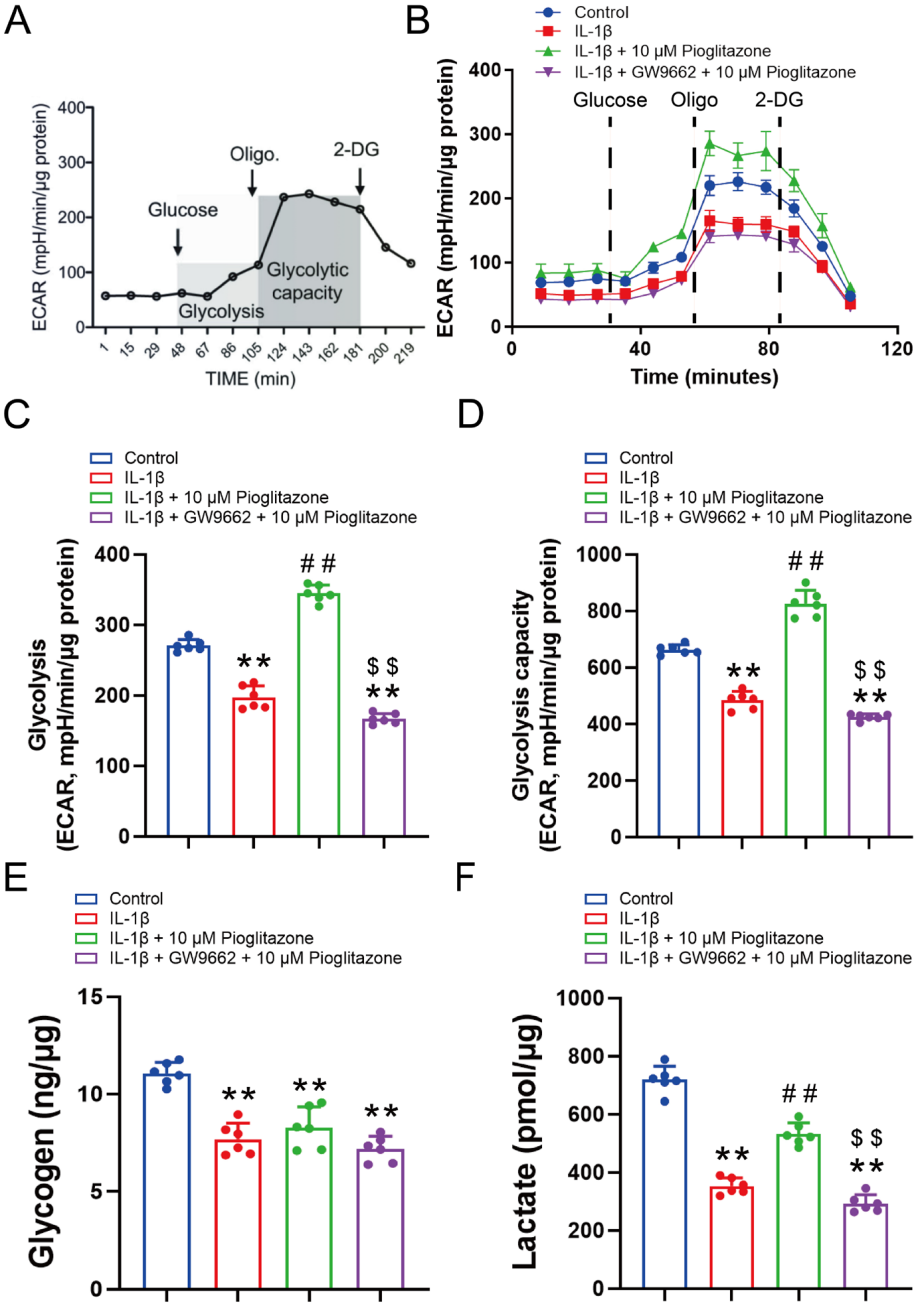
Pioglitazone reversed the suppression of glycolysis in OA chondrocytes. (A) Schematic illustration of the elements of glycolysis and glycolytic capacity dissected from the extracellular acidification rate (ECAR). (B–F) The ECAR curves (B), glycolysis (C: Area under curve of ECAR curves between glucose and oligomycin injection), glycolysis capacity (D: Area under curve of ECAR curves between oligomycin and 2‐Deoxy‐d‐glucose (2‐DG) injection), intracellular glycogen (E), levels of lactate of chondrocytes in the medium (F). ECAR was measured under glucose‐free conditions followed by the sequential adding of glucose (10 mM), oligomycin (Oligo, 1 μM), and 2‐DG (50 mM) as arrows indicated. Pioglitazone counteracted the IL‐1β‐induced inhibition of maximal ECAR and glycolytic capacity, whereas GW9662 negated the pro‐glycolytic effects of Pioglitazone. Each data point represents an ECAR measurement. Values are means ± SD of analyses through the one‐way ANOVA test (*n* = 6). *p < 0.05, **p < 0.01, versus control group; ^#^ *p* < 0.05, ^##^ *p* < 0.01 versus IL‐1β group; ^$^ *p* < 0.05, ^$$^ *p* < 0.01 versus IL‐1β + 10 μM Pioglitazone group.

PPAR‐γ is believed to play a role in Th2 cell differentiation, potentially by promoting glycolysis and oxidative phosphorylation [31]. Consequently, to evaluate the impact of Pioglitazone on chondrocyte glucose metabolism, the ECAR measured by Seahorse XF24 was employed to assess glycolytic capacity. Figure 3A illustrates the methodology for measuring glycolysis and glycolytic capacity in cells. In the control group, the administration of glucose (10 mM) stimulated glycolysis, resulting in an increase in the ECAR from 74.5 to 108.1 mpH·min^−1^·μg protein^−1^ (Figure 3B). The application of oligomycin, an inhibitor of ATP synthase, further elevated the ECAR to 225.9 ± 14.2 mpH·min^−1^·μg protein^−1^, approximately threefold higher than the baseline (Figure 3B). This suggests that chondrocytes underwent maximal anaerobic glycolysis to produce ATP. In the IL‐1β treated group, the ECAR values at baseline, following glucose application, and subsequent oligomycin treatment were 50.3 ± 4.2, 78.5 ± 4.2 and 159.3 ± 11.9 mpH·min^−1^·μg protein^−1^, respectively (Figure 3B). ECAR levels during glucose application followed by oligomycin application were significantly lower than those observed in the control group (Figure 3B). Notably, co‐incubation with Pioglitazone (10 μM) reversed the IL‐1β‐induced suppression of maximal ECAR, elevating it to approximately 1.2 times higher than the control (Figure 3B). However, this effect was negated by GW9662 (Figure 3B).

Further analysis of the area under the curve of the ECAR profile, partitioned into glycolysis and glycolytic capacity components, demonstrated that Pioglitazone co‐incubation effectively restored the suppressed glycolysis in the IL‐1β group (Figure 3C,D). The treatment with Pioglitazone may have augmented the quantity of glycolytic machinery. However, following the administration of GW9662, Pioglitazone failed to restore the pro‐glycolytic effect (Figure 3C,D).

In addition to glucose, glycogen serves as a significant source for lactate production. Analysis of cellular glycogen levels revealed that the IL‐1β group exhibited significantly lower glycogen content compared to the control group (Figure 3E). Furthermore, neither Pioglitazone nor GW9662 was able to elevate cellular glycogen concentrations, suggesting that Pioglitazone does not influence glycogen production (Figure 3E).

Subsequently, extracellular lactate levels were quantified. The extracellular lactate, serving as an indicator of lactate release, was significantly reduced in the IL‐1β group (Figure 3F). However, treatment with Pioglitazone effectively restored extracellular lactate levels (Figure 3F). Furthermore, in the GW9662 + Pioglitazone group, lactate concentrations were significantly lower compared to the Pioglitazone group alone (Figure 3F). These experimental results suggest that Pioglitazone can enhance glycolytic activity in IL‐1β‐induced OA chondrocytes, effectively reversing the suppression of glycolysis observed in these cells.

### 3.4 Pioglitazone Mitigated the Suppression of Chondrocyte Mitochondrial Oxygen Consumption Rate Induced by IL‐1β

**FIGURE 4:**
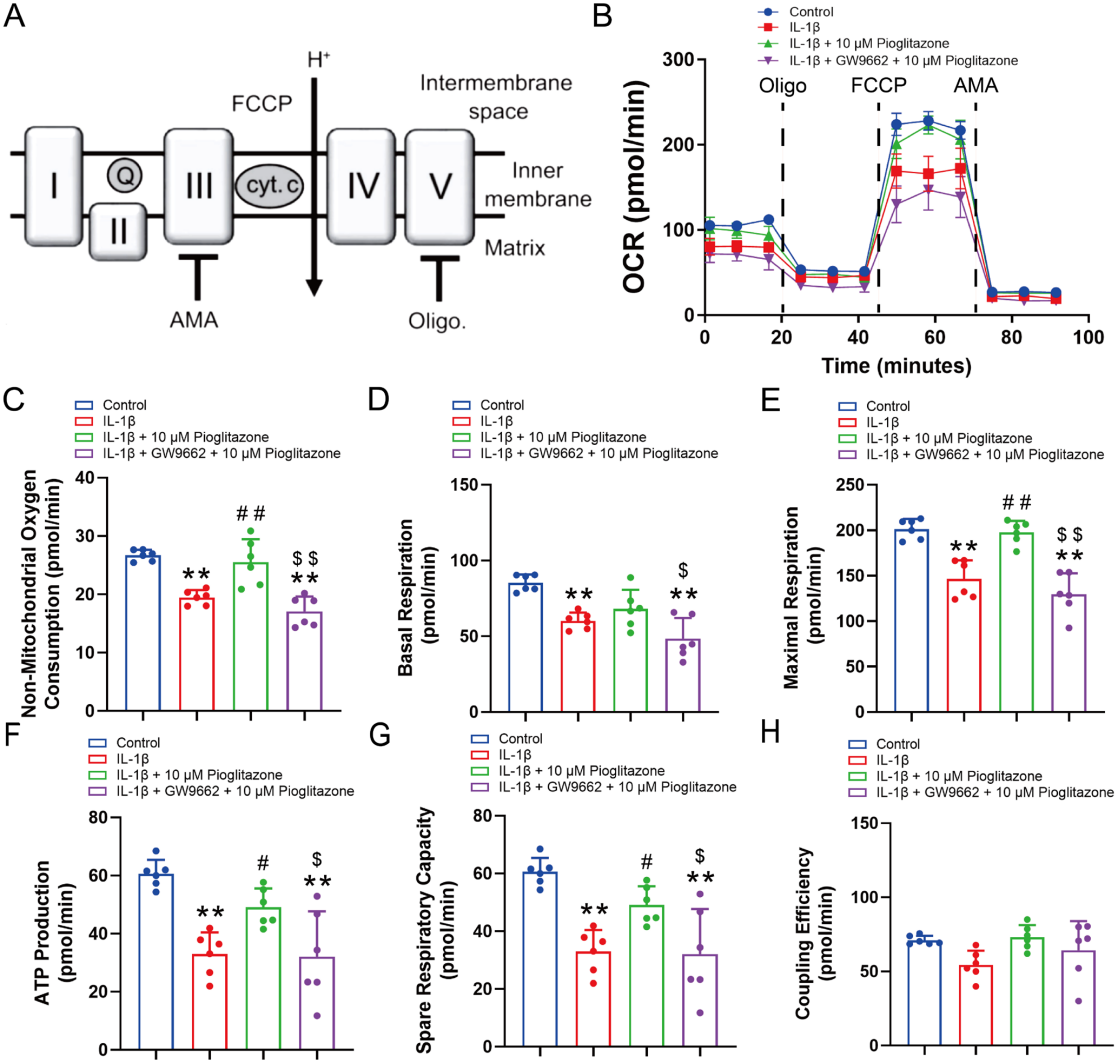
Pioglitazone reversed the suppressed chondrocyte mitochondrial oxygen consumption under IL‐1β. (A) schematic illustration of the elements of basal respiration, ATP production capacity, and maximal respiratory capacity dissected from the oxygen consumption rate (OCR). (B) The OCR curves (B), non‐mitochondrial oxygen consumption (C), basal respiration (D), maximal respiratory capacity (E), ATP production capacity (F), spare respiratory capacity (G), and the coupling efficiency (H) of chondrocytes. OCR was measured under basal conditions followed by the sequential addition of oligomycin (Oligo, 0.25 μM), FCCP (2 μM), and antimycin A (AMA; 1 μM). Pioglitazone mitigated the IL‐1β‐induced suppression of maximal respiration, ATP production, and respiratory capacity, while GW9662 diminished Pioglitazone's protective effects on oxidative phosphorylation. Each data point represents an OCR measurement. I, mitochondrial respiratory complex I (mt cpx I); II, mt cpx II; III, mt cpx III; IV, mt cpx IV; IV, mt cpx IV; cyt. c, cytochrome c; Q, coenzyme Q; Each data point represents a measurement. Values are means ± SD of analyses through the one‐way ANOVA test (*n* = 6). *p < 0.05, **p < 0.01, versus control group; ^#^ *p* < 0.05, ^##^ *p* < 0.01 versus IL‐1β group; ^$^ *p* < 0.05, ^$$^ *p* < 0.01 versus IL‐1β + 10 μM Pioglitazone group.

Subsequently, we investigated the impact of Pioglitazone on chondrocyte mitochondrial oxygen consumption rate (Figure 4A). Treatment with oligomycin reduced the OCR in the Control group to approximately 46% of the baseline rate, indicating that roughly 54% of chondrocyte oxygen consumption is associated with ATP synthesis (Figure 4B). FCCP, a mitochondrial uncoupler, was then applied. The application of FCCP dissipated the proton gradient across the inner mitochondrial membrane, resulting in an increase in OCR to approximately 204% of the baseline rate in the Control group (Figure 4B–H). The findings demonstrated that the maximum respiratory capacity of chondrocytes in the Control group was twice that of basal respiration (Figure 4B–H). In contrast, the IL‐1β group exhibited significant reductions in baseline respiration, maximal respiration following FCCP intervention, ATP production, and respiratory capacity reserve function compared to the Control group (Figure 4B–H). These observations suggest that IL‐1β impairs oxidative phosphorylation in chondrocytes. Importantly, treatment with Pioglitazone effectively restored maximal respiration, ATP production, and respiratory capacity reserve function (Figure 4B–H). Upon the addition of GW9662, oxidative phosphorylation in chondrocytes was reduced to levels comparable to those observed in the IL‐1β group. These findings indicate that Pioglitazone mitigated the IL‐1β‐induced inhibition of mitochondrial oxygen consumption rate in chondrocytes.

### 3.5 Pioglitazone Appears to Counteract the IL‐1β‐Mediated Suppression of Mitochondrial Electron Transport Activity in Chondrocytes

**FIGURE 5:**
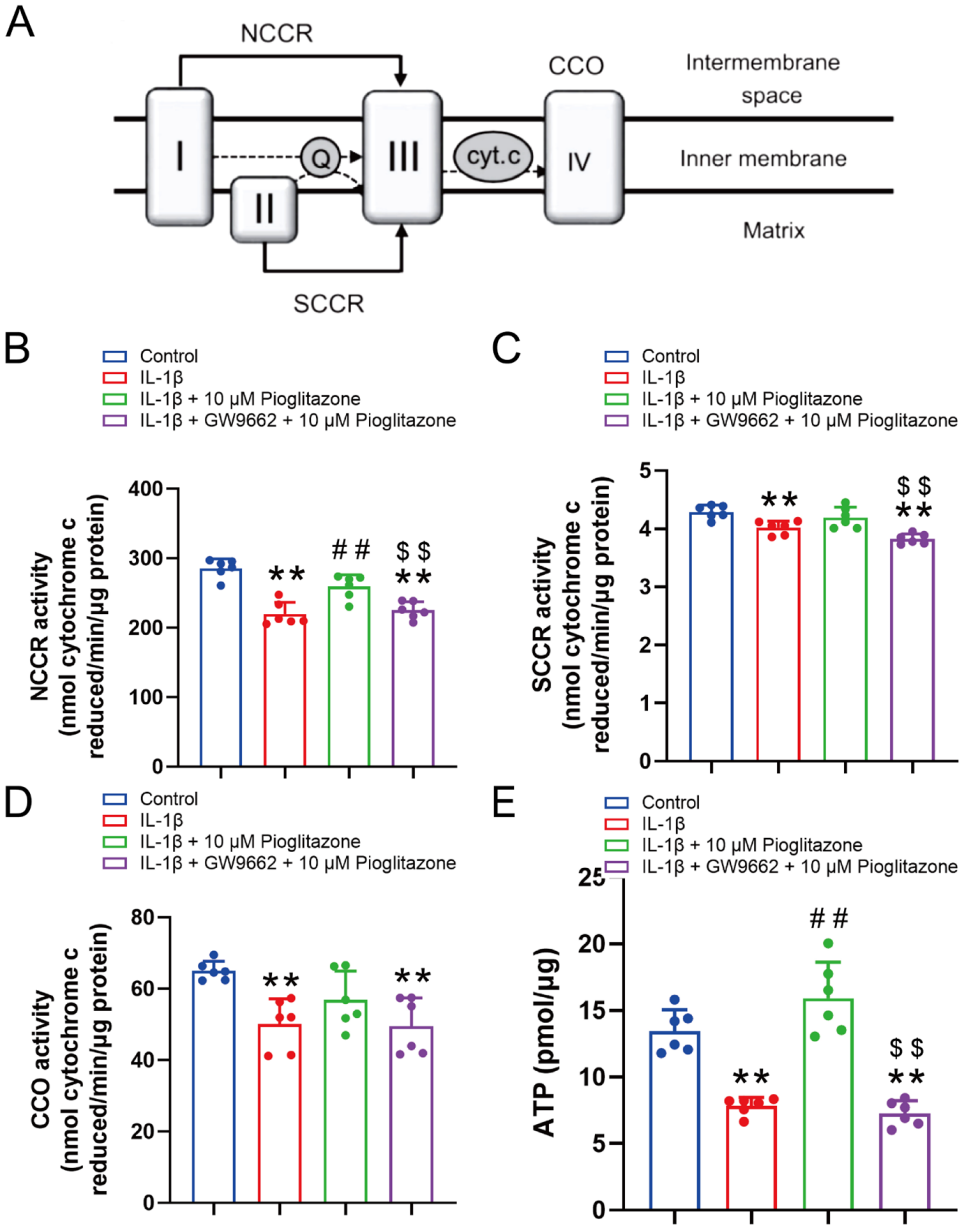
Pioglitazone appears to counteract the IL‐1β‐mediated suppression of mitochondrial electron transport activity in chondrocytes. (A) Schematic illustration of the activity of the NADH‐cytochrome c reductase (NCCR; electron transferred from complex I to III), the succinate cytochrome c reductase (SCCR; electron transferred from complexes II to III) and the cytochrome c oxidase (CCO; complex IV). (B–E) NCCR activity (B), SCCR activity (C), CCO activity (D), and ATP content (E) of chondrocytes. Pioglitazone alleviated the IL‐1β‐induced reduction of NCCR and ATP production, yet GW9662 annulled the effects of Pioglitazone. Each data point represents a measurement. Values are means ± SD of analyses through the one‐way ANOVA test (*n* = 6). *p < 0.05, **p < 0.01, versus control group; ^#^ *p* < 0.05, ^##^ *p* < 0.01 versus IL‐1β group; ^$^ *p* < 0.05, ^$$^ *p* < 0.01 versus IL‐1β + 10 μM Pioglitazone group.

Furthermore, the activities of enzymes involved in the electron transport chain (ETC) were assessed (Figure 5A). The activity of NADH cytochrome c reductase (NCCR), which facilitates the transfer of high‐energy electrons from NADH/NADPH to mitochondrial complex III via coenzyme Q, was compromised in the IL‐1β group (Figure 5B). Specifically, succinate cytochrome c reductase activity (SCCR, complex II) was found to be suppressed following IL‐1β intervention (Figure 5C). Similarly, the enzyme activity of cytochrome c oxidase (CCO), a crucial enzyme catalysing the final step in the mitochondrial electron transport chain and regulating oxidative phosphorylation, was suppressed in the IL‐1β group (Figure 5D). Additionally, ATP content was reduced in the IL‐1β group. These findings suggest that IL‐1β induces deficiencies in chondrocyte oxidative phosphorylation and inhibits energy production (Figure 5E). Furthermore, the reduced levels of NCCR and ATP were elevated following Pioglitazone treatment, without affecting SCCR or CCO activity within the electron transport chain (Figure 5B–E). However, the administration of GW9662 negated the effects of Pioglitazone (Figure 5B–E). These findings indicate that Pioglitazone counteracted the inhibitory impact of IL‐1β on mitochondrial electron transport activity in chondrocytes.

### 3.6 Pioglitazone Reversed the IL‐1β‐Induced Inhibition of GLUT1 Expression and Glucose Uptake in Chondrocytes

**FIGURE 6:**
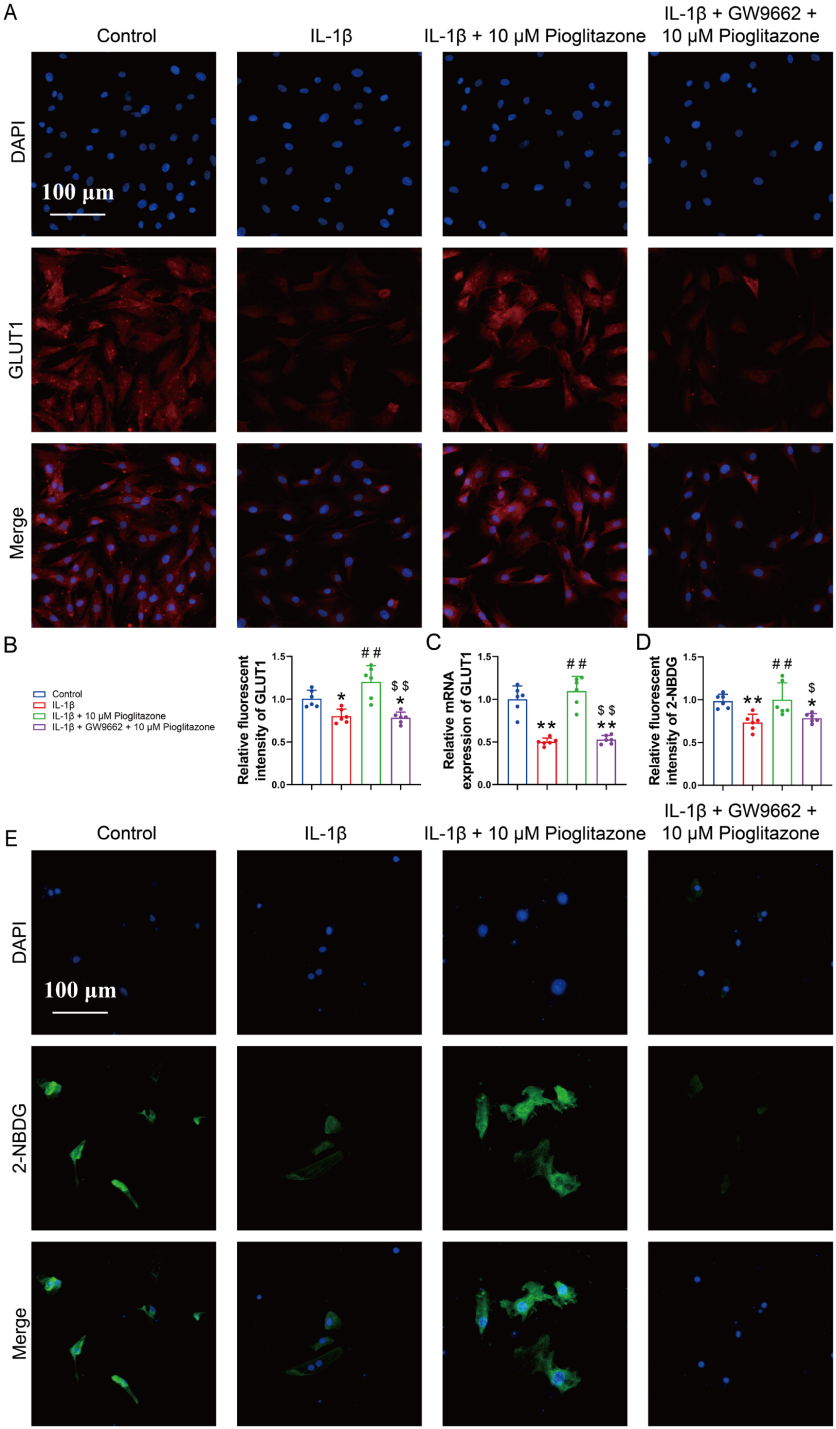
Pioglitazone reversed the IL‐1β‐induced inhibition of GLUT1 expression and glucose uptake in chondrocytes. (A, B) Expression of GLUT1 in chondrocytes was assessed and quantified by immunofluorescence staining. The fluorescence intensity of GLUT1 was reduced following GW9662 intervention. (C) RT‐qPCR analysis revealed that Pioglitazone was ineffective in upregulating the mRNA expression of GLUT1 in chondrocytes treated with GW9662. (D, E) The accumulation of 2‐[N‐(7‐nitrobenz‐2‐oxa‐1,3‐diazol‐4‐yl)amino]‐2‐deoxyglucose (2‐NBDG) was assessed and quantified by fluorescence staining. The fluorescence intensity of 2‐NBDG was decreased following GW9662 intervention. Each data point represents a measurement. Values are means ± SD of analyses through the one‐way ANOVA test (*n* = 6). *p < 0.05, **p < 0.01, versus control group; ^#^ *p* < 0.05, ^##^ *p* < 0.01 versus IL‐1β group; ^$^ *p* < 0.05, ^$$^ *p* < 0.01 versus IL‐1β + 10 μM Pioglitazone group.

GLUT1, the primary glucose transporter in chondrocytes, exhibited decreased expression in the IL‐1β‐treated group [32]. The immunofluorescence images and RT‐qPCR results further demonstrated a reduced expression of GLUT1 in the IL‐1β group (Figure 6A–C). Conversely, an increased presence of GLUT1 was observed in chondrocytes of the Pioglitazone group (Figure 6A–C). The incubation with GW9662 effectively counteracted the Pioglitazone‐induced upregulation of GLUT1 in chondrocytes (Figure 6A–C). These findings suggest the regulation of GLUT1 expression in chondrocytes.

Subsequently, the basal level of glucose uptake was evaluated by measuring intracellular 2‐NBDG in chondrocytes. Immunofluorescence images revealed a decreased uptake of 2‐NBDG in chondrocytes of the IL‐1β group compared to the Control group (Figure 6D,E). Furthermore, Pioglitazone reinstated the fluorescence intensity of 2‐NBDG in the absence of GW9662, indicating that Pioglitazone ameliorated the impaired glucose uptake capacity in chondrocytes (Figure 6D,E).

### 3.7 Pioglitazone Exerts Chondroprotective Effects in OA Mice

**FIGURE 7:**
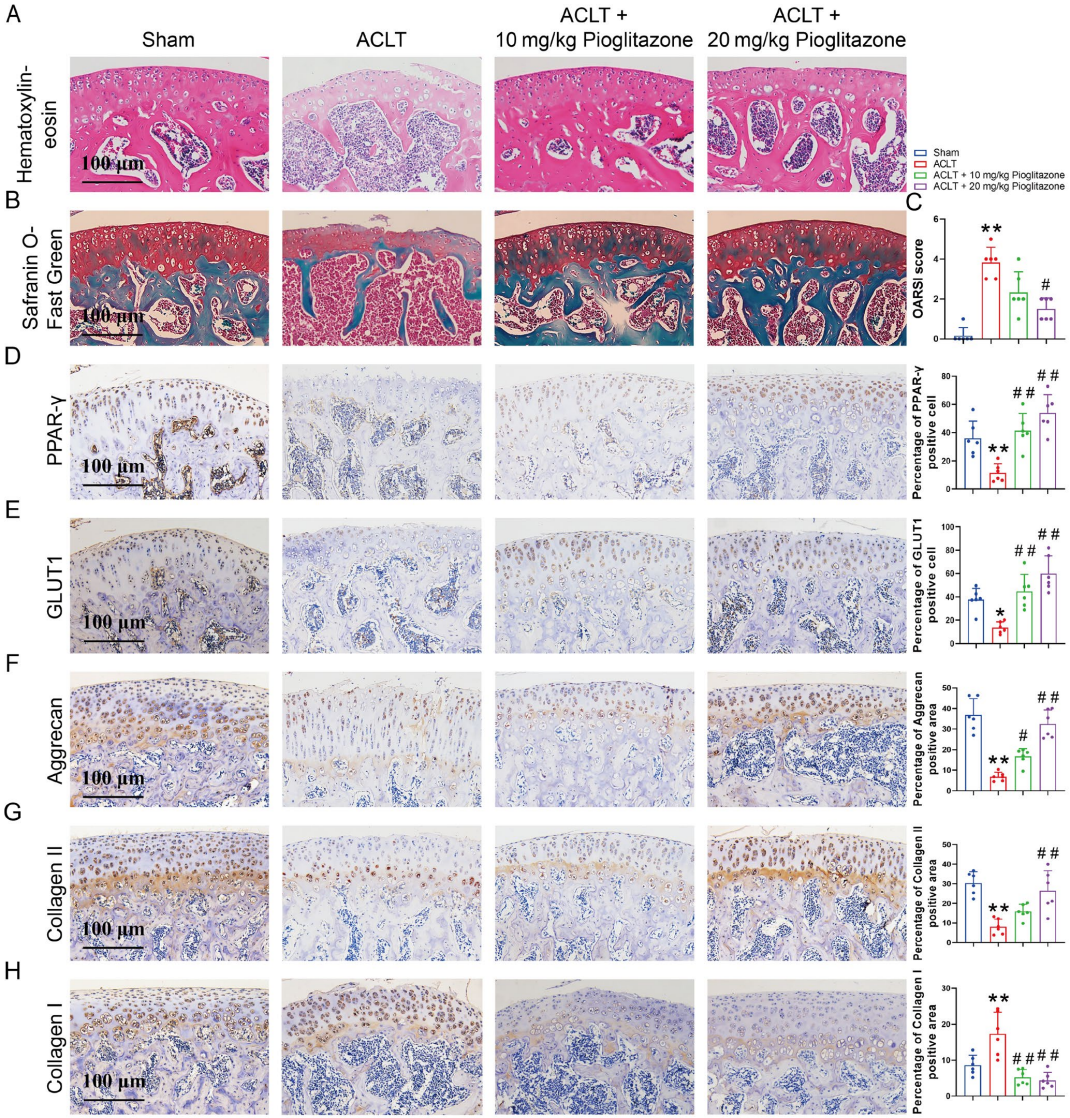
Pioglitazone exerts chondroprotective effects in OA mice. (A) Haematoxylin and eosin staining demonstrated that Pioglitazone alleviated articular cartilage abrasion and fissures in OA mice. (B) Safranin O‐Fast Green staining indicated that Pioglitazone reduced articular cartilage degradation in OA mice. (C) The OARSI score was used to determine the extent of cartilage deterioration. Grade 0 was for intact surface and cartilage; Grade 1 for intact surface only; Grade 2 for surface discontinuity, Grade 3 for vertical fissures; Grade 4 for erosion, Grade 5 for denudation, and Grade 6 for deformation. Treatment with Pioglitazone decreased the OARSI scores in OA mice. (D–H) Assessment of the protein expression of PPAR‐γ, GLUT1, aggrecan, collagen II, and collagen I in articular cartilage by immunohistochemical staining. Pioglitazone treatment increased PPAR‐γ, GLUT1, aggrecan, and collagen II expression, alongside decreased collagen I expression in OA mice. Each data point represents a measurement. Values are means ± SD of analyses through the one‐way ANOVA test (*n* = 6). *p < 0.05, **p < 0.01, versus control group; ^#^ *p* < 0.05, ^##^ *p* < 0.01 versus IL‐1β group; ^$^ *p* < 0.05, ^$$^ *p* < 0.01 versus IL‐1β + 10 μM Pioglitazone group.

To elucidate the role of Pioglitazone in vivo, we established an OA mouse model via ACLT and administered Pioglitazone through intra‐articular injection. Haematoxylin and eosin staining revealed that ACLT modelling induced articular cartilage abrasion and fissures in the mice (Figure 7A). Safranin O‐Fast Green staining indicated that ACLT modelling contributed to articular cartilage degradation and elevated OARSI scores (Figure 7B,C). Conversely, intervention with Pioglitazone mitigated articular cartilage wear in mice, which was associated with a reduction in OARSI scores (Figure 7A–C).

Subsequently, we investigated the underlying mechanisms through immunohistochemical staining. The ACLT group exhibited reduced expression of PPAR‐γ, GLUT1, aggrecan, and collagen II, alongside increased collagen I expression, indicating chondrocyte metabolic disorders and phenotypic loss (Figure 7D–H). In contrast, the Pioglitazone group exhibited a reversal of the aforementioned adverse phenomena induced by ACLT (Figure 7D–H).

## 4 Discussion

OA is a degenerative joint disease marked by the destruction of articular cartilage and inflammation of the synovium [33]. It is characterised by pathological changes in joint architecture, including cartilage degeneration, synovial inflammation, and subchondral sclerosis with osteophyte formation [8]. Factors such as age, obesity, mechanical injuries, and heredity significantly contribute to the development of OA. Nonetheless, the pathogenesis of OA remains unclear [34]. The contemporary management of OA primarily encompasses both surgical and non‐surgical interventions [35]. Surgical treatments, however, carry risks such as joint loss and infections, along with substantial financial costs [36]. Non‐surgical approaches include pharmacologic therapy, appropriate exercise, weight management, and physical therapy [37]. Pharmacologic interventions involve the oral and local administration of medications, as well as intra‐articular injections. Oral non‐steroidal anti‐inflammatory drugs and opioids can be efficacious in alleviating OA‐related pain [38]. However, prolonged use of these medications may lead to gastrointestinal and cardiovascular complications, as well as renal and hepatic damage [38]. Topical administration of non‐steroidal anti‐inflammatory drugs or herbal remedies may offer anti‐inflammatory and analgesic benefits; however, adverse effects such as localised rashes, burning sensations, and pruritus can occasionally manifest [39]. For patients who exhibit inadequate responses to oral and topical therapies, intra‐articular irrigation and injection techniques may be employed to deliver medications directly to the lesion site, thereby alleviating pain and enhancing joint function [40]. Nonetheless, it is important to note that repeated treatments may result in damage to the tendons and ligaments of the articular cartilage [41]. Therefore, it is imperative to develop alternative pharmacological treatments for OA that demonstrate efficacy while minimising adverse effects.

Current research indicates that metabolic disorders significantly contribute to the pathogenesis of OA, and that metabolic regulation is integral to its management [42]. Unlike most tissues, articular cartilage is devoid of blood vessels, nerves, and lymphatic vessels; instead, it relies on synovial fluid within the joints for nutrient supply, with glycolysis serving as the primary energy source for chondrocytes [43]. Glycolytic metabolic disturbances can induce chondrocyte hypertrophy and extracellular matrix degradation, thereby facilitating the progression of OA [44]. In osteoarthritic joints or inflammatory environments, low‐grade inflammation results in chondrocyte hypoxia, prompting metabolic reprogramming that augments glycolytic pathways to meet the energy demands of chondrocytes [45]. The glycolysis metabolite, lactic acid, inhibits both metabolic reprogramming and proinflammatory signalling pathways [46]. Consequently, targeted modulation of glycolytic processes is anticipated to enhance chondrocyte viability and mitigate OA progression. In this study, we employed Pioglitazone, a well‐established pharmacological agent for diabetes management, to investigate its potential impact on chondrocyte metabolism, with the aim of generating novel insights for OA treatment.

Pioglitazone functions as a specific PPAR‐γ agonist and insulin sensitiser, commonly prescribed for patients with diabetes mellitus or metabolic syndrome [18, 19]. PPAR‐γ is known to modulate the effector functions of human T helper 9 cells by enhancing glycolysis [47]. Additionally, in the context of neuroinflammatory and neurodegenerative diseases, Pioglitazone has been shown to augment cellular glucose metabolism and mitochondrial activity [20]. Pioglitazone facilitates the normalisation of chondrocyte oxidative phosphorylation in mice subjected to a high fructose diet [21]. In our study, molecular docking analysis revealed that Pioglitazone exhibits a high affinity for PPAR‐γ. Furthermore, RT‐qPCR and immunohistochemical staining demonstrated that Pioglitazone enhances the expression of PPAR‐γ both in vivo and ex vivo. Subsequent investigations into cellular energy metabolism in chondrocytes under various conditions—including normal, inflammatory, post‐Pioglitazone treatment, and post‐PPAR‐γ inhibition—indicated that Pioglitazone significantly restored inhibited glycolysis and oxidative phosphorylation in OA chondrocytes. Additionally, Pioglitazone was found to promote normal energy metabolism and ATP production.

The promotive effect of PPAR‐γ on energy metabolism has been substantiated by various studies [48]. In human T helper 9 cells, PPAR‐γ enhances glycolysis to sustain cellular functions [47]. Additionally, PPAR‐γ activation augments macrophage glycolysis and promotes M1 polarisation [49]. Activation of PPAR‐γ through multiple mechanisms enhances regulatory T cell function by promoting fatty acid oxidation [50]. Furthermore, catalpol improves insulin sensitivity and mitochondrial respiration in the skeletal muscle of T2DM mice via the activation of PPAR‐γ and insulin signalling pathways [51]. Furthermore, PPAR‐γ enhances cardiac mitochondrial function and mitigates mitochondrial damage [52]. Our study demonstrated that Pioglitazone promoted glucose uptake and glycolysis in chondrocytes via activation of PPAR‐γ and facilitated mitochondrial electron transport activity and oxidative phosphorylation. Conversely, the metabolic function of chondrocytes was significantly inhibited following the administration of GW9662, an antagonist of PPAR‐γ.

Abnormal chondrocyte metabolism appears to be a response to alterations in the inflammatory microenvironment and may play a pivotal role in cartilage degeneration and the progression of OA [45]. In healthy joints, chondrocytes typically maintain metabolic equilibrium [53]. However, under environmental stress, chondrocytes adapt to microenvironmental changes by altering their metabolic pathways [44]. Similar metabolic adaptations are observed in other joint cells, such as synoviocytes [54]. These shifts in metabolic pathways are associated with mitochondrial dysfunction, increased anaerobic glycolysis, and changes in lipid and amino acid metabolism [42]. Investigating the role of chondrocyte metabolism in OA elucidates critical aspects of disease pathogenesis. Alterations in chondrocyte metabolism may delineate distinct OA phenotypes. In our animal experiments, we observed a reduction in the expression of aggrecan and collagen II, alongside an increase in collagen I expression in chondrocytes within the cartilage tissues of OA‐afflicted mice. These findings suggest a deviation from the normal chondrocyte phenotype. Conversely, treatment with Pioglitazone appeared to preserve the normal chondrocyte phenotype. To the best of our knowledge, this study represents the first investigation into the effects of Pioglitazone on chondrocyte energy metabolism and its potential therapeutic implications for OA. The findings from this research are expected to contribute significantly to the development of novel pharmacological agents targeting metabolic pathways of therapeutic relevance.

Nonetheless, this study is subject to certain limitations. Primarily, due to the challenges associated with procuring human chondrocytes, our experiments were conducted exclusively on mouse chondrocytes. Furthermore, although chondrocytes are the predominant cell type in articular cartilage, it is crucial to consider the microenvironmental alterations within the joint capsule. Therefore, future research should also focus on elucidating the effects of Pioglitazone and PPAR‐γ on synoviocyte metabolism.

## Author Contributions

**Jiaqi Shi:** data curation (lead), investigation (equal), methodology (equal), software (lead), writing – original draft (lead). **Tianlun Gong:** investigation (equal), methodology (equal), writing – review and editing (equal). **Yi Zhou:** conceptualization (lead), funding acquisition (lead), writing – review and editing (equal).

## Ethics Statement

All animal experiments were approved by the Hubei Provincial Animal Care and Use Committee.

## Consent

The authors have nothing to report.

## Conflicts of Interest

The authors declare no conflicts of interest.

## Data Availability

The datasets used and/or analysed during the current study are available from the corresponding author on reasonable request.
